# Inhibition of NOX2 reduces locomotor impairment, inflammation, and oxidative stress after spinal cord injury

**DOI:** 10.1186/s12974-015-0391-8

**Published:** 2015-09-17

**Authors:** Guzal Khayrullina, Sara Bermudez, Kimberly R. Byrnes

**Affiliations:** Anatomy, Physiology and Genetics Department, Uniformed Services University, Room B2048, 4301 Jones Bridge Road, Bethesda, MD 20814 USA

**Keywords:** NOX2, Polarization, Microglia, Inflammation, Spinal cord injury, Oxidative stress

## Abstract

**Background:**

Spinal cord injury (SCI) results in the activation of the NADPH oxidase (NOX) enzyme, inducing production of reactive oxygen species (ROS). We hypothesized that the NOX2 isoform plays an integral role in post-SCI inflammation and functional deficits.

**Methods:**

Moderate spinal cord contusion injury was performed in adult male mice, and flow cytometry, western blot, and immunohistochemistry were used to assess NOX2 activity and expression, inflammation, and M1/M2 microglia/macrophage polarization from 1 to 28 days after injury. The NOX2-specific inhibitor, gp91ds-tat, was injected into the intrathecal space immediately after impact. The Basso Mouse Scale (BMS) was used to assess locomotor function at 24 h post-injury and weekly thereafter.

**Results:**

Our findings show that gp91ds-tat treatment significantly improved functional recovery through 28 days post-injury and reduced inflammatory cell concentrations in the injured spinal cord at 24 h and 7 days post-injury. In addition, a number of oxidative stress markers were reduced in expression at 24 h after gp91ds-tat treatment, which was accompanied by a reduction in M1 polarization marker expression.

**Conclusion:**

Based on our findings, we now conclude that inhibition of NOX2 significantly improves outcome after SCI, most likely via acute reductions in oxidative stress and inflammation. NOX2 inhibition may therefore have true potential as a therapy after SCI.

## Background

Spinal cord injury (SCI) is followed by an acute but long-lasting inflammatory response, marked by invasion of blood-borne cells and activation of endogenous cells and a marked increase in reactive oxygen species (ROS) production. Neutrophils are the first cells to respond, invading the spinal cord within minutes to hours and populating the injured tissue for days [1]. Following this response is a macrophage/microglial response that typically peaks within 4–7 days [2]. Both of these cell types express the NADPH oxidase (NOX) 2 enzyme, which is a primary producer of ROS in the spinal cord after injury.

Research has shown that this macrophage/microglia response is not homogenous, and both cell types can be polarized into pro-inflammatory M1 cells or anti-inflammatory M2 cells [3]. Early work demonstrated that, after SCI, the M1/M2 ratio is roughly equal acutely after injury but shifts to an M1 dominant response within a few days [4]. It is currently unclear what signals result in this M1/M2 shift, although some research has suggested that the NOX enzyme may play a role in not only this polarization [5] but also general inflammatory responses and oxidative stress.

The NOX enzyme complex is a primary source of ROS, with seven currently known family members, including NOX2 [6]. The enzyme functions by transferring electrons from NADPH inside the cell, across the membrane, and then to extracellular oxygen, generating a superoxide. Components of the NOX2 enzyme, including the enzymatic core, gp91^PHOX^, and membrane-bound companion protein, p22^PHOX^, are elevated after SCI [7–11], and NOX activity is upregulated for at least 6 months post-injury [9].

The NOX2-specific inhibitor, gp91ds-tat, a 20-amino acid peptide that interferes with the assembly of activated NOX2, has been used in research for a number of different disease states, both in vitro and in vivo designs. In vitro, gp91ds-tat effectively reduces the release of ROS from activated microglia [12]. Previously, we found that systemic administration of gp91ds-tat reduced both oxidative stress and cytokine production acutely after SCI [11]. In addition, gp91ds-tat was found to be neuroprotective in a traumatic brain injury model [13].

The aim of the current paper was to explore the effect of specific inhibition of NOX2 on oxidative stress, inflammation, and function after SCI. We now show that inhibition of NOX2 reduces post-injury inflammation and improves locomotor recovery in mice. In addition, we show that NOX2 inhibition shifts microglial/macrophage polarization toward the M2 phenotype, which may have significant neuroprotective effects in the injured spinal cord.

## Methods

### Animal handling and surgical methods

Adult male C57Bl6 mice were utilized in all experiments (20–25 g, Taconic Farms, Derwood, MD). Mice were group housed and received food and water ad libitum with a 12:12 h light cycle. A total of 111 male mice were used for this study; 13 mice were removed from the study due to post-surgical complications. All experiments complied fully with the principles set forth in the “Guide for the Care and Use of Laboratory Animals” and were approved by the Uniformed Services University IACUC.

 All subjects undergoing surgery received isoflurane (Primal Healthcare, Andhra Pradesh, India). Mice received a laminectomy, followed by a contusion simulating moderate SCI using the Infinite Horizons Impactor (50 kdyn; Precision Systems and Instrumentation, Fairfax Station, VA). Mice were immediately given an intrathecal injection of either gp91ds-tat or scrambled ds-tat (AnaSpec, Inc., Fremont, CA) diluted to 50 μM in saline in a 5 μl volume at the lesion epicenter. After expelling the liquid, the needle was held under the dura for 30 s prior to removal. The incision was then closed, and animals were maintained on heating pads until mice regained movement. Acetaminophen (Children’s Tylenol, 200 mg/kg) was added to drinking water for 72 h post-injury. Manual bladder expression was performed daily until normal bladder expression returned. Naïve mice did not undergo surgery or receive isoflurane.

### Functional testing

The Basso Mouse Scale (BMS) was used to rate locomotor function and recovery. Injured mice in recovery were scored in seven categories including ankle movement, plantar placement, stepping, coordination, paw position, trunk instability, and tail position. Mice (*n* = 9/group) from the 28-day time point group were observed at 24 h by two investigators blinded to the treatment group and then weekly post-injury.

### Immunohistochemistry

At 2 h (*n* = 4 gp91ds-tat, 3 scrambled ds-tat), 24 h (*n* = 4 gp91ds-tat, 4 scrambled ds-tat), 7 days (*n* = 3 gp91ds-tat, 4 scrambled ds-tat), and 28 days (*n* = 4 gp91ds-tat, 4 scrambled ds-tat), injured or naïve (*n* = 4) mice were anesthetized (Euthasol, 0.22 ml/kg, IP) and perfused with 100 ml of 0.9 % sterile saline, followed by 300 ml of 10 % buffered formalin phosphate (Fisher Scientific, Fair Lawn, NJ). A 5-mm spinal cord segment, 2.5 mm caudal and 2.5 mm rostral to the injury site, was extracted. Spinal cords were kept in formalin for 24 h and then transferred to a 30 % sucrose solution. Spinal cords were then cut into 20-μm axial sections. Standard fluorescent immunohistochemistry was performed with primary antibodies that had been previously characterized in the laboratory [11, 12], including Iba1 (1:100, Wako), CD86 (1:200, Abcam), Mannose Receptor/CD206 (1:50, Abcam), and 3NT (1:5, Abcam). Alexa-Fluor secondary antibodies (Invitrogen) were used for visualization. Slides were coverslipped using mounting media containing DAPI to counterstain for nuclei (Vector Labs, Burlingame, CA).

Immunofluorescence was detected and photographed in the dorsal column region within the 5-mm region of interest using an Olympus DP72 microscope with Olympus cellSens microscopy software (Olympus, Center Valley, PA) or NanoZoomer Digital Pathology system (Hamamatsu Photonics, K.K., Japan). For 3NT immunohistochemistry, fluorescent immunohistochemistry was quantified as previously described using pixel density measurement in Scion Image [14]. For all other immunohistochemistries, images were qualitatively evaluated for comparison to flow cytometry and western blotting results. For all stains, at least five sections taken from regular intervals within the 5-mm region of interest were evaluated.

### Western blot

At 24 h (*n* = 4 gp91ds-tat, 4 scrambled ds-tat), 7 days (*n* = 6 gp91ds-tat, 5 scrambled ds-tat), and 28 days (*n* = 3 gp91ds-tat, 3 scrambled ds-tat) post-injury or after no intervention (naïve, *n* = 8), mice were euthanized and tissue was flushed with 100 ml of 0.9 % sodium chloride. A 5-mm spinal cord segment, 2.5 mm rostral and 2.5 mm caudal to the lesion epicenter, was collected and protein extracted with RIPA (1×) buffer (Thermo Scientific, Rockford, IL). Aliquots of 25 μg were used for western analysis using the following primary antibodies: CD86 (Abcam, 1:250), CD206 (Abcam, 1:500), iNOS (Cell Signaling Technology, 1:200), and phospho-p47^PHOX^ (p-p47; Sigma, 1:250). Immune complexes were detected with appropriate secondary antibodies and chemiluminescence reagents (Pierce, Rockford, IL). GAPDH was used as a control for gel loading and protein transfer. ImageJ software was used to quantify bands.

### Oxyblot

All samples from the western blot experiments were also used for oxyblot experiments. Millipore OxyBlot Protein Oxidation Detection Kit (Temecula, CA) was used according to the manufacturer’s instructions. β-actin (Abcam) was used as control for gel loading and protein transfer. ImageJ software was used to quantify resultant bands.

### Flow cytometry

A 5-mm spinal cord segment, 2.5 mm caudal and 2.5 mm rostral to the injury site, was processed for flow cytometry at 24 h (*n* = 4 gp91ds-tat, 4 scrambled ds-tat) and 7 days (*n* = 8 gp91ds-tat, 9 scrambled ds-tat) post-injury, following a perfusion with 100 ml of 0.9 % saline. Cells were isolated using a 70, 37, 30 % Optiprep gradient (Axis-Shield, Wesbury, NY) in 1× HBSS solution. Cells were blocked with TruStain fcX (1:200 BioLegend, San Diego, CA), and macrophages, microglia, lymphocytes, neutrophils, and dendritic cell populations were immunolabled with PE CD11b (1:200 eBioscience Inc., San Diego, CA), APC/Cy7 CD45(1:500, BioLegend, San Diego, CA), APC CD3ε (1:200, BioLegend), and FITC GR-1(1:2000 BioLegend, San Diego, CA) in flow cytometry staining buffer. Sytox Blue was used as a dead cell marker (1:1000 Molecular Probes, Inc. Eugene, OR). OneComp eBeads (one drop per sample, eBioscience Inc., San Diego, CA) were used in place of tissue as marker controls, while tissue was used for unstained control for analysis purposes. Corresponding isotype controls were used per fluorochrome. Populations of interest were gated on, and at least 200,000 cells were collected for each sample. Data was analyzed using FlowJo software (FlowJo, LLC, Ashland, OR).

### Statistics

Quantitative data are presented as mean ± standard error of the mean. BMS and BMS subscore data were obtained by two blinded investigators and analyzed using repeated measures ANOVA with Bonferroni’s multiple comparisons test. All other quantitative data were analyzed using unpaired *t* test or one-way ANOVA as appropriate. All statistical tests were performed using the GraphPad Prism Program, Version 6.03 for Windows (GraphPad Software, San Diego, CA). A *p* value <0.05 was considered statistically significant.

## Results

### NOX2 inhibition improves locomotor function

**Fig. 1:**
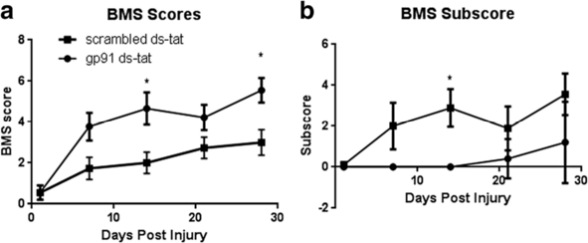
Inhibition of NOX2 improves motor function. Gross hindlimb motor function was scored using the BMS (**a**), with further analysis of hindpaw function with the BMS subscore (**b**). Mice were tested at days 1, 7, 14, 21, and 28 post-injury. Those treated with gp91ds-tat showed significant increases in BMS score and BMS subscore at 7 days; this improvement was sustained through 28 days in the BMS score. *Points* represent mean ± SEM. *N* = 9/group. *p < 0.05, repeated measures ANOVA

The BMS score provides a general locomotion score reflecting all four limbs, while the subscore reflects more distinct measures of locomotor control, such as plantar stepping, coordination, and paw position. Although both animal groups exhibited a gradual recovery, mice treated with gp91ds-tat showed an overall improvement with a significant improvement at 14- and 28-day time points compared to those treated with scrambled ds-tat (Fig. 1a). Furthermore, gp91ds-tat-treated mice demonstrated a recovery in more fine motor control, as shown in the BMS subscore, after 7 days with a significant improvement at 14 days (Fig. 1b). Scrambled ds-tat-treated mice only demonstrated an increase in BMS subscore at 28 days post-injury.

### NOX2 inhibition reduces acute oxidative stress

**Fig. 2:**
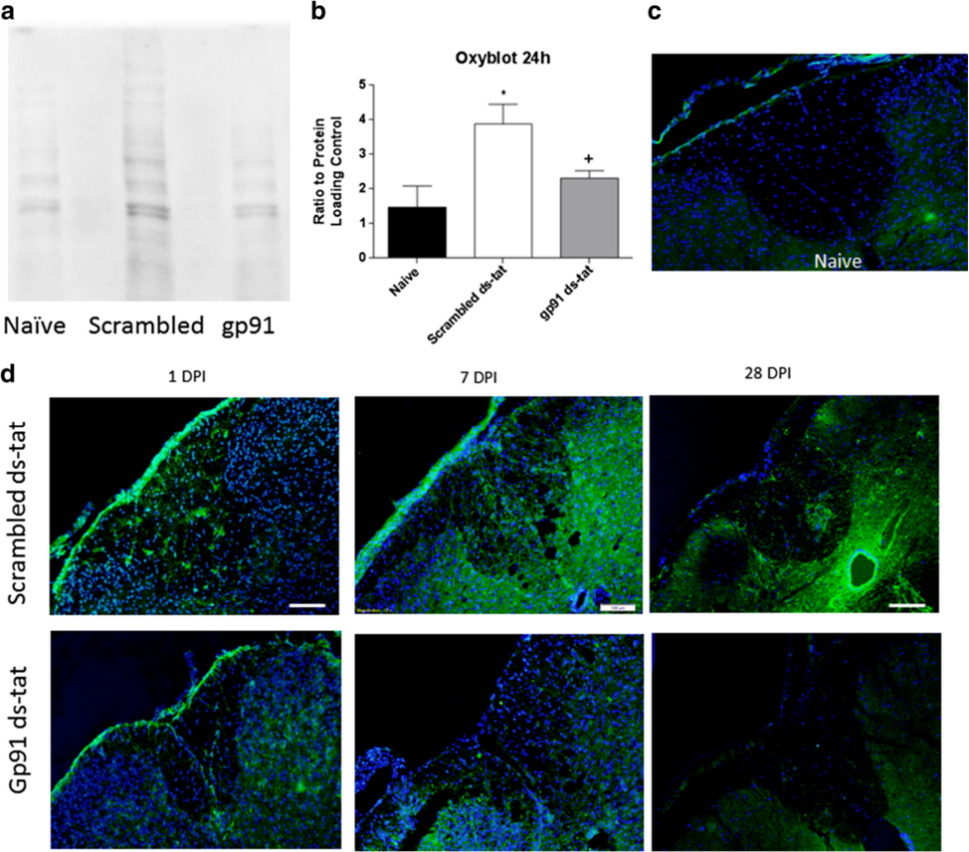
Acute inhibition of NOX2 reduces markers of oxidative stress. OxyBlot Protein Oxidation assay was used to detect protein carbonylation in naïve (*n* = 8), scrambled ds-tat- (*n* = 4), and gp91ds-tat-treated mice (*n* = 4) at 24 h post-injury (**a**). Densitometry demonstrated a significant increase in protein carbonylation in scrambled ds-tat-treated mice, which was significantly reduced to naïve levels with gp91ds-tat treatment (**b**). At these same time points, spinal cord tissue was immunolabeled for 3-nitrotyrosine (*green*), a marker of nitrosylated protein (scrambled ds-tat: *n* = 4/time point; gp91ds-tat: *n* = 4/24 h, 3/7 days; 4/28 days). DAPI nuclear stain is shown in *blue*. Naïve (*n* = 4) tissue is shown in **c**. Qualitative analysis shows that scrambled ds-tat-treated tissue had elevated 3NT immunolabeling in comparison to naïve tissue, primarily in gray matter (**d**). This elevation grew through 7 days and was lessened at 28 days. Treatment with gp91ds-tat appeared to reduce this immunolabeling at all time points. Bar = 100 μm. *p < 0.05 vs naïve; +*p* < 0.05 vs scrambled; one-way ANOVA with Tukey’s post-test. *Bars* represent mean ± SEM

An oxyblot assay, which detects carbonylated proteins, was performed to measure the overall oxidative stress present in the injured tissue (Fig. 2a). While injury resulted in an increase in carbonylated proteins at 24 h, carbonylation of proteins was significantly reduced in tissue that received gp91ds-tat in comparison to the scrambled ds-tat (Fig. 2b).

To further investigate oxidative stress, tissue was stained with an antibody against 3NT, a marker for nitrotyrosine-containing proteins (Fig. 2c, d). At 24 h post-injury, scrambled ds-tat-treated tissue demonstrated elevated 3NT immunostaining in both white and gray matters in comparison to naïve tissue. Qualitatively, gp91ds-tat-treated spinal cord showed less staining at both 24 h and 7 days post-injury. At 28 days post-injury, residual expression in the lesion site was still observed in both groups, although reduced in comparison to earlier time points. When the immunostain was quantified, no significant differences were observed between the gp91ds-tat and scrambled ds-tat groups at any time point, although a trend toward significance was seen at all time points, with slight reductions in 3NT quantity in the white matter.

**Fig. 3:**
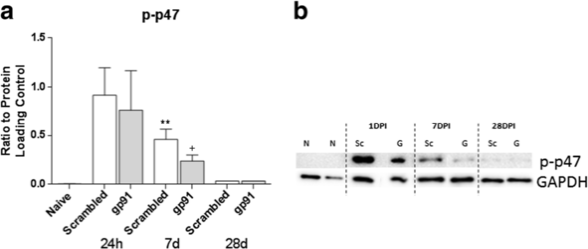
Acute inhibition of NOX2 using gp91ds-tat reduces p47^PHOX^ expression and/or phosphorylation. Phosphorylated p47^PHOX^ (p-p47) and GAPDH were evaluated at 24 h, 7 days, and 28 days post-injury in naïve (*N*; *n* = 8), scrambled ds-tat- (*S*; *n* = 4/24 h; 6/7 days; 3/28 days), and gp91ds-tat-treated samples (*G*; *n* = 4/24 h; 5/7 days; 3/28 days). Bands were observed at 47 and 38 kDa, respectively, in a representative western blot (**a**). Pixel densitometry for p-p47^PHOX^ (**b**) showed significant induction of phosphorylation of p47^PHOX^ at 24 h. NOX2 inhibition reduced p-p47^PHOX^ levels at 7 days to a point that was not significantly greater than sham. **p < 0.01 vs naïve. +*p* < 0.01 vs scrambled; one-way ANOVA with Tukey’s post-test. *Bars* represent mean ± SEM

Finally, to determine whether NOX2 inhibition may interfere with NOX2 activity, phosphorylation of the NOX2 component p47^PHOX^ was assessed. Gp91ds-tat interferes with the activation of the NOX2 enzyme by blocking binding of phosphorylated p47^PHOX^ with the gp91^PHOX^ subunit [6]. The expression of this enzyme is dependent on a feed-forward cycle, wherein ROS production by NOX2 can activate downstream signal transduction pathways that increase NOX2 component expression, including p47^PHOX^. Therefore, expression of phosphorylated p47^PHOX^ was assessed using western blotting (Fig. 3b). Phosphorylation of p47^PHOX^ was markedly elevated at 24 h post-injury, with no significant difference between groups. However, by 7 days post-injury, phosphorylation of p47^PHOX^ was significantly reduced in the gp91ds-tat-treated group, suggesting that activity of this enzyme was reduced at this time point, despite the lack of acute reduction (Fig. 3a).

### NOX2 inhibition reduces inflammatory cell populations in the injured spinal cord

**Fig. 4:**
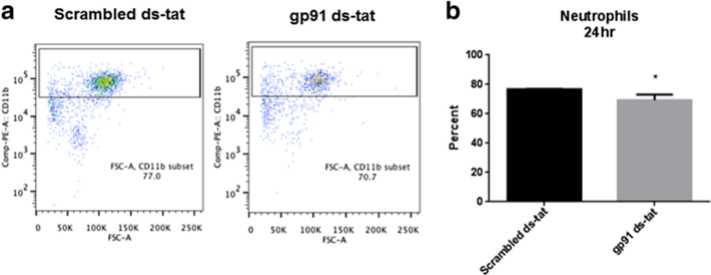
Acute inhibition of NOX2 reduces neutrophil populations by 24 h post-injury. Neutrophils were characterized as alive, CD45^+^/CD11b^+^/GR-1^+^ population (**a**). **b** Quantitation shows that gp91ds-tat treatment significantly reduced this population. *p < 0.05 vs scrambled ds-tat, Student’s *t* test. *Bars* represent mean ± SEM

**Fig. 5:**
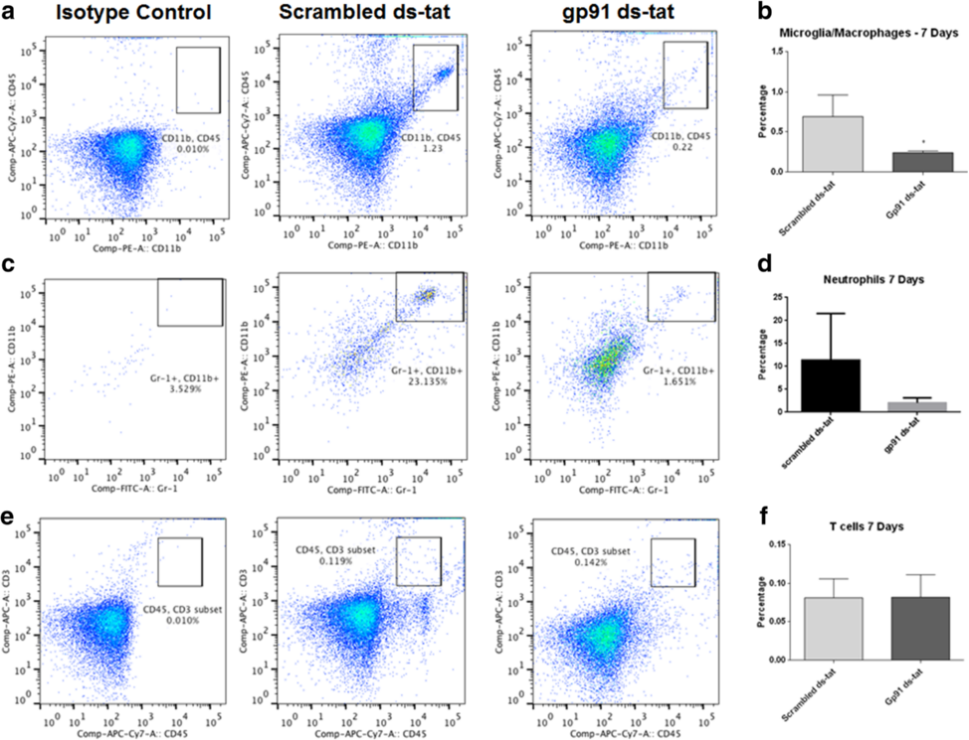
Acute NOX2 inhibition reduces inflammatory response by 7 days post-injury. All listed cell types were under a live CD45+ gate. Microglia/macrophage were further isolated as CD11b^+^Gr-1^−^ (**a**), neutrophils as CD11b^+^Gr-1^+^ (**c**), and T cells as CD3^+^ (**e**). The *first column* in each row indicates an isotype control. Quantitation shows that gp91ds-tat treatment significantly reduced the macrophage/microglia (**b**) and neutrophil (**d**) populations but had no significant effect on the T cell population (**f**). *p < 0.05 vs scrambled ds-tat, Student’s *t* test. *Bars* represent mean ± SEM

**Fig. 6:**
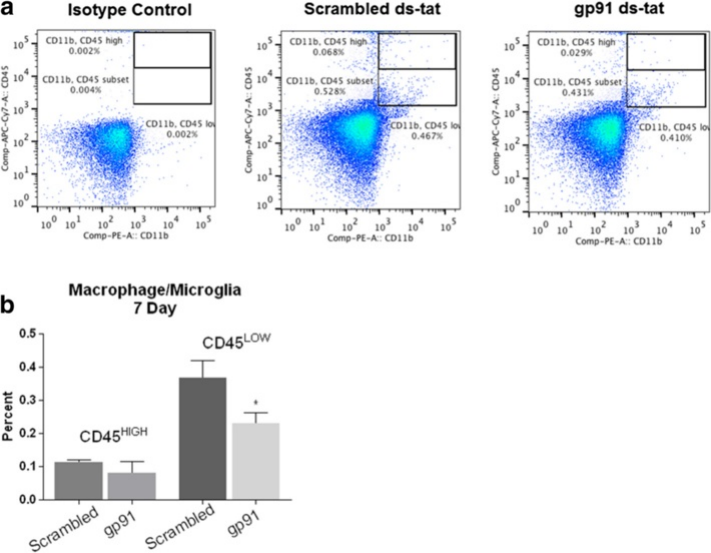
Acute NOX2 inhibition specifically targets microglia. Flow cytometry was used to analyze macrophage and microglia populations in the injured region. Live cells were first gated on and further differentiated based on CD11b^+^ and CD45^+^. CD45 high, macrophages, and CD45 low, microglia, were arbitrarily chosen by a researcher blinded to study (**a**). Quantitation demonstrated no significant difference in macrophages at 7 days post-injury between scrambled ds-tat- and gp91ds-tat-treated tissue. Microglia, on the other hand, showed a significant decrease in tissue treated with gp91ds-tat compared to scrambled ds-tat (**b**). *p < 0.05 vs scrambled ds-tat, Student’s *t* test. *Bars* represent mean ± SEM

In order to determine the mechanism behind the improved functional outcome and determine the influence of reduced NOX2 activity and oxidative stress, flow cytometry was used to assess the percentages of macrophage, microglia, lymphocyte, and neutrophil populations at 24 h and 7 days post-injury. At 24 h post-injury, gp91ds-tat treatment was found to significantly reduce the neutrophil population (CD45^+^/GR-1^+^; Fig. 4b). By 7 days, macrophage/microglia (CD45^+^/CD11b^+^/GR-1^−^; Fig. 5b) was significantly reduced by gp91ds-tat administration. Neutrophils had a trend toward lower expression, although no significance was found (Fig. 5d). Further separation of the macrophage/microglia population by dividing this group into CD45^high^ (macrophage) and CD45^low^ (microglia) expression groups demonstrated that at 7 days post-injury, there was a greater proportion of microglia than macrophages in the injured spinal cord, and that this population demonstrated a greater response to gp91ds-tat treatment (Fig. 6b). No significant difference in T cell population (CD45^+^/CD3^+^; Fig. 5f) was noted between treatment groups.

**Fig. 7:**
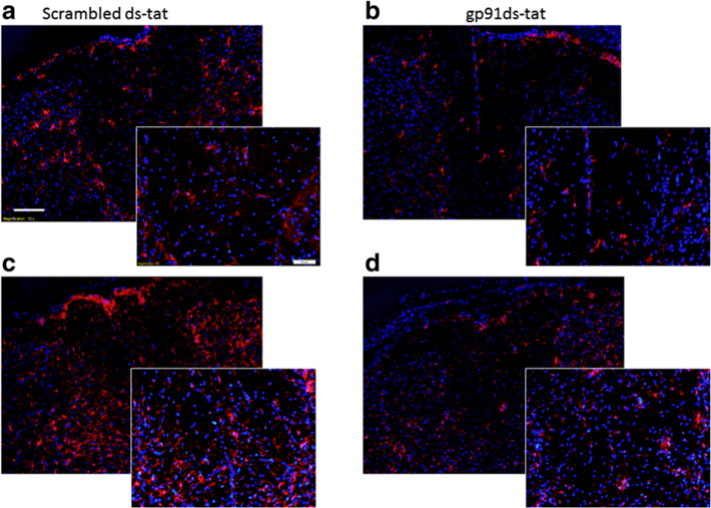
Acute NOX2 inhibition reduces microglia/macrophage presence in injured spinal cord tissue. Gp91ds-tat- and scrambled ds-tat-treated tissue at 24 h (**a**, **b**; *n* = 4/group) and 7 days (**c**, **d**; *n* = 3 gp91ds-tat, 4 scrambled ds-tat) post-injury was stained for the pan microglia/macrophage marker Iba1 (*red*). DAPI nuclear stain is shown in *blue*. At 24 h post-injury, the scrambled ds-tat-treated tissue (**a**) have a greater number of activated microglia compared to gp91ds-tat-treated tissue as seen in ×10 magnification (**b**). The activated morphology includes a more amoeboid body with retracted dendrites (which can be better observed in the higher magnification *inset image* (×20)). At the same time point, the microglia present in gp91ds-tat tissue have long processes and smaller bodies. This difference is greater at the 7-day time point when microglia are at their peak (**c**, **d**). The gp91ds-tat-treated microglia at 7 days (×20) are activated, but the number are still downregulated compared to those treated with scrambled-tat. Lower magnification and high-magnification images shown; bar (×20) = 50 μm; bar (×10) = 100 μm

We next confirmed these flow cytometry results using immunohistochemistry. Immunostaining demonstrated a marked elevation in Iba1 staining in scrambled-tat-treated tissue from 24 h to 7 days post-injury (Fig. 7a–d). In tissue that had received gp91ds-tat administration, this elevation was not observed, and Iba1 staining was relatively low throughout the study.

### NOX2 inhibition shifts microglia/macrophage polarization

As gp91ds-tat was found to shift the inflammatory response, it was unclear if this had any effect on microglia/macrophage polarization. Previous studies have shown that NOX2 inhibition can alter polarization states [5]. We therefore assessed polarization markers in the spinal cord using flow cytometry, immunohistochemistry, and western blotting.

**Fig. 8:**
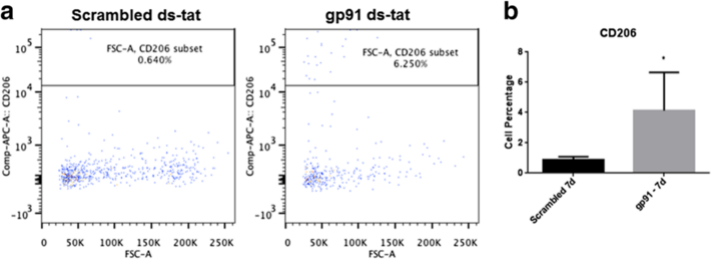
Acute NOX2 inhibition increases the M2 marker CD206 by 7 days post-injury. Cells were gated on live CD45^+^CD11b^+^Gr-1^−^CD206^+^ population (**a**). Quantitation shows that gp91ds-tat treatment significantly increased the CD206 population (**b**). *p < 0.05 vs scrambled ds-tat, Student’s *t* test. *Bars* represent mean ± SEM

**Fig. 9:**
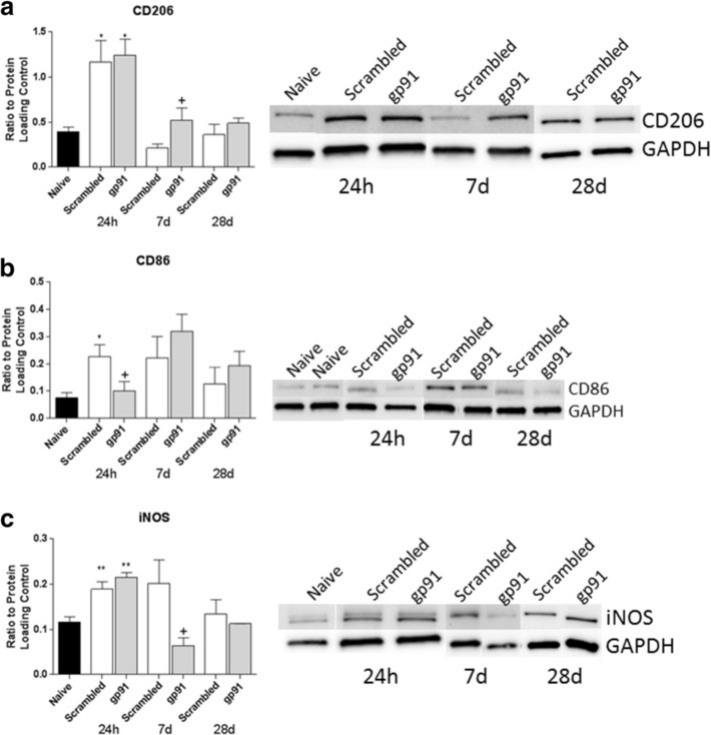
Acute inhibition of NOX2 using gp91ds-tat alters microglia/macrophage polarization marker expression. Protein samples (25 μg) were probed for CD206, iNOS, CD86, and GAPDH at 24 h, 7 days, and 28 days post-injury in naïve (*n* = 8), scrambled ds-tat- (*n* = 4/24 h; 6/7 days; 3/28 days), and gp91ds-tat-treated samples (*n* = 4/24 h; 5/7 days; 3/28 days). Pixel densitometry for CD206 (**a**), CD86 (**b**), and iNOS (**c**) showed significant alteration with gp91ds-tat treatment in comparison to scrambled ds-tat treatment. The M2 marker CD206 was elevated in both groups at 24 h in comparison to naïve, but remained elevated only in the gp91ds-tat-treated group at 7 days post-injury, before returning to baseline levels by 28 days. In contrast, at 24 h, the inhibition of NOX2 significantly reduced the expression of the M1 marker CD86, although by 7 and 28 days, no significant difference was observed between groups. Finally, a second M1 marker, iNOS, was elevated in both groups at 24 h post-injury, and gp91ds-tat treatment only prevented this induction by 7 days post-injury. *p < 0.05 vs naïve; +*p* < 0.05 vs scrambled; one-way ANOVA with Tukey’s post-test. *Bars* represent mean ± SEM

At 7 days post-injury, the M2 marker CD206 showed a significant increase with gp91ds-tat treatment compared to scrambled ds-tat, using both flow cytometry (Fig. 8) and western blotting (Fig. 9a).

Western blot data from 24 h post-injury revealed that expression of the M1 marker CD86 was significantly reduced in the gp91ds-tat group in comparison to the scrambled ds-tat group (Fig. 9b). However, expression of this protein then returned to levels demonstrated in the scrambled ds-tat-treated group by 7 and 28 days post-injury. An additional M1 marker, iNOS, demonstrated significantly reduced expression at 7 days post-injury, with no significant difference between groups at 24 h and 28 days (Fig. 9c).

## Discussion

We now show that a single acute central administration of the NOX2 inhibitor gp91ds-tat led to significant improvements in a number of measures of recovery after moderate SCI. Recovery was seen on both the functional and cellular levels, including increases in BMS score and subscore, reductions in measures of oxidative stress, and reduced inflammatory cell presence through 7 days post-injury. Despite the observation that this single acute treatment did not induce inflammatory effects beyond 7 days, functional improvement continued through at least 28 days, indicating the importance of acute and subacute interventions.

Although the NADPH complex enzyme group encompasses several different NOX and DUOX complexes, our use of gp91ds-tat was specific to the NOX2 isoform. This peptide contains a sequence that mimics gp91^PHOX^ and binds to phosphorylated p47^PHOX^ [15]. This peptide then blocks ROS production specifically by the NOX2 isoform, having no effect on NOX1 or 4 [16]. In vitro, gp91ds-tat has been shown to block the assembly of the NOX2 enzyme [17]. In vivo, gp91ds-tat reduced neuronal death in a brain injury model when administered as a single pre-treatment dose [13].

A number of NOX inhibitors have been used in the past. These include apocynin, which interferes with the assembly of the active NOX enzyme [18], and diphenyleneiodonium (DPI), which directly blocks the catalytic activity of NOX (and other flavoprotein-containing enzymes) [19]. Apocynin has been shown to reduce oxidative stress in the central nervous system during conditions including sepsis [20] and brain injury [13, 21], providing significant neuroprotection. More importantly, apocynin administration following SCI in rats significantly reduced a number of inflammatory and oxidative stress markers, including neutrophil invasion, nitrotyrosine production, and pro-inflammatory cytokine expression [22]. Further, DPI administration reduced oligodendrocyte and oligodendrocyte precursor death in lipopolysaccharide induced inflammation models [23]. More importantly, we have shown that DPI administration following SCI reduces lesion volume and post-injury inflammation [9]. However, DPI and apocynin have been found to have numerous non-specific effects and can act on enzymes beyond the NOX family that contain flavoproteins [24]. While picomolar concentrations have been shown to reduce these flavoprotein effects [25], these drugs are still not specific for any particular NOX isoform. In contrast, we now show that specific and acute inhibition of the NOX2 isoform can reduce inflammation in both the acute and subacute periods, leading to long-term functional improvement.

The functional improvement observed was measured using both the BMS and the BMS subscore, suggesting improvements in both gross and fine motor skills. These improvements first became significant at 14 days post-injury, although a trend was observed as early as 7 days, which is the time point at which significant differences in several markers of oxidative stress and inflammation were observed. Despite the fact that no significant difference in oxidative stress marker or inflammation was observed at 28 days post-injury, functional improvement continued through 28 days, suggesting that acute inhibition of NOX2 was sufficient to result in sustained functional changes. However, it is important to note that by 21–28 days post-injury, the significance in the BMS subscore between the gp91ds-tat- and scrambled ds-tat-treated groups was lost, suggesting that sustained or even delayed administration of treatment may have further benefits.

A number of studies have shown that NOX isoforms are upregulated after central nervous system injury, including brain injury [12, 13] and SCI [7–11], and may contribute to post-injury oxidative stress. This study adds to that literature, demonstrating elevated protein carbonylation and nitrosylation in combination with elevated expression and/or phosphorylation of p47^PHOX^. Phosphorylation of the p47^PHOX^ subcomponent results in unmasking of PHOX-binding domains in the protein, allowing for assembly and activation of the enzyme [26]. Interestingly, we found the greatest elevation in protein expression of the NOX2 component at 24 h after injury, with expression and phosphorylation reduced by 7 days and returning to baseline levels by 28 days. This is in contrast to our previous work, wherein gene and protein expression of NOX2 components gp91^PHOX^ and p22^PHOX^ as well as NOX activity were elevated for months after injury [9]. However, the extended expression profile was determined in a rat model of SCI; it is likely that the mouse model used in the current study demonstrates a different NOX expression and activity profile. Future research will continue to explore these differences. Regardless, the evidence indicates that NOX2 is active for at least 7 days post-injury, suggesting that a delayed treatment approach may be efficacious in this model and others.

Concurrently or following this alteration in oxidative stress status, we observed a significant reduction in post-injury inflammation, including acute and subacute reductions in neutrophil invasion and subacute reductions in macrophage/microglial populations with gp91ds-tat administration. Interestingly, microglial populations, defined by low expression of the CD45 marker, were most affected by gp91ds-tat administration. This is likely due to the local rather than systemic administration of the NOX2 inhibitor. Systemic administration of apocynin, a non-specific NOX inhibitor, has been shown to similarly reduce inflammatory responses, but without specific effects on microglia [22]. Further, this study demonstrated that apocynin administration reduced adhesion molecule expression, which may provide an explanation for the reduction in neutrophil invasion observed with local spinal cord injection of gp91ds-tat. In addition, while analysis with both flow cytometry and immunohistochemistry demonstrated no significant difference in microglia/macrophage presence at 24 h, qualitative analysis suggested a morphological change, with fewer “activated” macrophages/microglia at this time point in gp91ds-tat-treated tissue. Activated microglia/macrophages are known to demonstrate an amoeboid structure with retracted dendrites, which was seen in tissue that received the scrambled ds-tat control treatment; gp91ds-tat-treated tissue demonstrated a qualitatively greater presence of ramified cells, with small cell bodies and long thin processes [27].

To further explore the phenotype of the affected macrophages and microglia, cellular polarization was investigated. In 2009, Kigerl et al. [4] demonstrated that there is a balance of M1 and M2 markers acutely after injury that is followed by an increase in the M1:M2 ratio in the subacute period. Our data support these findings, with elevated M1 and M2 marker expression at acute time points and a shift toward M1 dominance by 7 days post-injury. Gp91ds-tat administration appears to limit this shift, however, reducing the decline of the M2 polarization marker at 7 days post-injury (Figs. 8 and 9a) and reducing M1 markers at 1 or 7 days post-injury (Fig. 9b, c). The influence of NOX2 inhibition on M1 marker expression was somewhat variable, with acute effects observed in the CD86 marker and subacute effects in the iNOS marker; this may reflect a variability in polarization phenotypes over time. Future research is needed to more precisely identify the influence of NOX inhibition on polarization. Further, it should be noted that M1 and M2 marker expression in the gp91ds-tat-treated group had returned to scrambled ds-tat-treated group levels by 28 days post-injury, suggesting that a single acute administration is insufficient to retain a long-lasting alteration in polarization.

Despite this, we, and others, have now shown that acute alterations in microglial polarization and NOX activity can influence each other. Administration of the M2-polarizing cytokine IL4 induced a reduction in NOX2 expression by 24 h [28]. Inhibition of NOX activity, via knockout of p47^PHOX^ and gp91^PHOX^ or administration of apocynin, on the other hand, significantly reduced M1 polarization and shifted microglia in LPS-treated brain tissue to an M2 polarization state [5]. This shift was accompanied by a reduction in pro-inflammatory cytokines and Iba1 immunolabeling. These results are supported by our current study and extended to the spinal cord trauma field, demonstrating that inhibition of the NOX2 isoform specifically reduces M1 polarization, increases M2 polarization, and reduces a number of pro-inflammatory markers.

In conclusion, these data demonstrate that a single acute NOX2-specific inhibition can have dramatic effects on oxidative stress, inflammation, and microglial polarization following SCI, and that these effects significantly improve post-injury functional recovery. NOX2, therefore, plays an integral role in post-injury inflammation, and specific activity on this enzyme can be targeted in order to help avoid non-target effects. Despite the marked alterations, we did observe a return to untreated injury levels in many of our outcome measures by 1 month post-injury, suggesting that work remains to elucidate the appropriate treatment approach in order for this therapy to be considered for further development and clinical use.
